# Real-World Long-Term Remission Maintenance for 10 Years With Thiopurines in Ulcerative Colitis

**DOI:** 10.1093/crocol/otab003

**Published:** 2021-02-10

**Authors:** Satohiro Matsumoto, Hirosato Mashima

**Affiliations:** Department of Gastroenterology, Saitama Medical Center, Jichi Medical University, Saitama, Saitama, Japan

**Keywords:** thiopurines, immunomodulators, maintaining remission, ulcerative colitis

## Abstract

**Background:**

To evaluate the therapeutic outcomes and long-term prognosis of patients receiving remission maintenance therapy using thiopurines for ulcerative colitis (UC).

**Methods:**

Of 193 biologic-naive patients with UC who began thiopurine therapy at our hospital between 2000 and 2019, 161 patients were included after the exclusion of 32 patients who were intolerant to thiopurines and discontinued the drugs within 3 months. Short- and long-term clinical outcomes were retrospectively analyzed. Subsequently, the patients were divided into 2 groups (exacerbation and nonexacerbation groups) and clinical outcomes were analyzed and compared. Multivariate analysis was performed to identify risk factors for UC exacerbation. Finally, adverse events observed in 193 patients were examined.

**Results:**

Clinical remission rates at 2 months, 6 months, and 1 year after the start of thiopurine therapy were 50.0%, 58.0%, and 63.9%, respectively. At 1, 2, 5, and 10 years, the cumulative event-free rates were 77.6%, 60.8%, 48.5%, and 42.2%, respectively; the cumulative UC exacerbation rates were 17.0%, 32.5%, 42.2%, and 43.7%, respectively; and the cumulative colectomy rates were 0.6%, 1.3%, 8.5%, and 10.7%, respectively. Prior use of steroids (dose ≥40 mg/d) was a significant risk factor for UC exacerbation during remission maintenance therapy with thiopurines (hazard ratio, 2.26; 95% confidence interval, 1.18–4.34; *P* = 0.014). Adverse reactions occurred in 42 patients (21.8%; 46 events). Concurrent diseases were observed in 18 patients (9.3%).

**Conclusions:**

Thiopurines were effective for long-term maintenance of remission in steroid-dependent/refractory UC. Their effect weakened in only a few patients continuously treated with them for 4 years or longer.

## INTRODUCTION

Ulcerative colitis (UC) is a chronic inflammatory colonic disease; currently, the causes remain unknown. In Japan, the number of patients with UC has been increasing every year. In 2014, the number of registered patients reached 180,000. Furthermore, according to the “Nationwide Epidemiological Survey” conducted by the Science Research Group of the Ministry of Health, Labour and Welfare in 2016, the number had reached 220,000. Many patients with UC require steroids to induce remission. Some patients experience difficulty withdrawing from steroids, even after successful remission induction with steroid therapy, while others have difficulty achieving improvement despite steroid therapy. Of patients with UC treated with steroids, 16% are steroid-resistant, and 22% become steroid-dependent after 1 year of treatment.^1^ Many patients who are difficult to treat are either steroid-dependent or steroid-refractory. While the therapeutic strategies for these patients have long been a major issue, thiopurines have been used as a treatment aimed at steroid withdrawal and long-term remission maintenance. In addition, antitumor necrosis factor (TNF) α agents^2–4^ and, recently, vedolizumab,^5^ ustekinumab,^6^ and tofacitinib^7^ as well as immunomodulators have been used in Japan for the induction and maintenance of remission in patients with steroid-dependent/refractory UC, and the efficacy of these drugs has been evaluated. The use of biologics for inflammatory bowel disease (IBD) is steadily increasing. Because they are much more expensive than conventional drugs, the economic impact of biologics on health care has been an issue in recent years.^8^ Thus, one of the important issues regarding remission maintenance therapy for patients with UC who are steroid-dependent appears to be how well to use thiopurines before the start of biologic therapy without careful consideration. The present study aimed to evaluate the therapeutic outcomes and long-term prognosis of patients with UC who underwent remission maintenance therapy with thiopurines after remission induction with mesalazine, steroids, tacrolimus, and cytapheresis. Very few studies have examined the long-term efficacy of thiopurines for the treatment of UC. Under the current circumstances, where biologics and molecular target drugs are becoming the mainstays of treatment for steroid-dependent/refractory UC, only a few studies on thiopurines are expected to be reported in the future. In this regard, the present study appears to be extremely important.

## MATERIALS AND METHODS

### Subjects

This was a retrospective single-center study conducted at our hospital. Of 787 Japanese patients with UC who visited our hospital between 2000 and 2019, 252 patients who received thiopurine therapy [azathioprine (AZA) or 6-mercaptopurine (6-MP)] were enrolled in the present study, and their data were extracted from medical records. The exclusion criteria were as follows: (1) patients who had started thiopurine therapy at another hospital (n = 44), (2) patients who started thiopurine therapy in combination with anti-TNFα agents, were receiving the agents, or had been treated with them (n = 15), and (3) patients who discontinued thiopurines within 3 months of thiopurine therapy because of intolerance (n = 32). Ultimately, 161 patients [92 men and 69 women; mean age (±standard deviation) at onset, 33 ± 16 years; mean disease duration (±standard deviation), 6.2 ± 7.1 years] were included.

UC was diagnosed based on the characteristic endoscopic and biopsy findings, after excluding other possible inflammatory bowel disorders. Steroid treatment history included steroids given orally, as enemas, or by injection (both short and long term). Steroid dependence was defined as recurrence during the tapering of the steroid dose, and steroid refractoriness was defined as no response to prednisolone administered at a dose of 1–1.5 mg/kg/d for 1–2 weeks.

### Treatment Protocol for AZA

Thiopurines are thiopurine derivatives and have an inhibitory effect on nucleic acid synthesis and drugs with an immunomodulatory action. In Japan in 2006, the national health insurance system began covering AZA for steroid-dependent UC, whereas 6-MP for UC is not yet being covered by the insurance system. For patients included in this study, AZA or 6-MP was administered for thiopurine therapy. AZA was typically administered, and 6-MP was used for patients who were intolerant to AZA. The doses of the thiopurines were adjusted to obtain a white blood cell (WBC) count of 3–5 × 10^9^/L.

### Efficacy Evaluation

In 161 patients, the clinical activity index (CAI), clinical remission rate, and response rate were evaluated at baseline and at 2 months, 6 months, and 1 year after the start of thiopurine therapy; blood test results (leukocyte, neutrophil, hemoglobin, mean corpuscular volume platelet, C-reactive protein) were evaluated before and after the start of thiopurine therapy. Clinical symptoms were scored using the CAI^9^ developed by Lichtiger et al. The components of the CAI include the following: bowel movement frequency (score 0–4), nocturnal diarrhea (score 0–1), blood in stool (score 0–3), fecal incontinence (score 0–1), use of antidiarrheal drugs (score 0–1), abdominal pain (score 0–3), general well-being (score 0–5), and abdominal tenderness (score 0–3). Clinical remission was defined as a CAI of 3 or less, and clinical response was defined as a decrease in scores by 50% or more from baseline.^10^

Patients were followed up until June 2020. The cumulative event-free rate, cumulative UC exacerbation rate, and cumulative colectomy rate were retrospectively analyzed and evaluated. Next, the 161 patients were divided into 2 groups: the exacerbation group, which required additional treatment (eg, steroid, cytapheresis, biologics, among others) due to UC exacerbation during thiopurine therapy; and the nonexacerbation group, which required no additional treatment. The background factors, treatment contents, and clinical courses were analyzed and evaluated. Furthermore, multivariate analysis was performed to identify risk factors for UC exacerbation. Finally, subanalyses were performed in 193 patients [109 men and 84 women; mean age at onset (±standard deviation), 34 ± 16 years; mean disease duration (±standard deviation), 6.1 ± 6.8 years], including 32 patients who were excluded from the main analyses because of one of the exclusion criterion (ie, discontinuation of thiopurines due to intolerance within 3 months of thiopurine therapy). Adverse events occurring during the follow-up period were assessed. Events included (1) the discontinuation of thiopurines due to intolerance to or complications from thiopurines, (2) exacerbation requiring additional treatment (eg, steroid, cytapheresis), (3) the start of biologic therapy, (4) hospitalization due to UC exacerbation, and (5) colectomy. For thiopurines, the cumulative continuation rate and cumulative event-free rate were analyzed.

### Ethical Considerations

This study was approved by The Etiological Study Ethical Review Board of [Saitama Medical Center, Jichi Medical University]. Because we produced and used anonymized data, informed consent was not needed.

### Statistical Analysis

Data are expressed as the mean ± standard deviation or as a percentage. Statistical analyses were performed using either a Student *t* test, Fisher exact test, or a Friedman test with the Bonferroni method. The cumulative rates were evaluated using the Kaplan–Meier method and compared using the log-rank test. Predictive factors were analyzed using multivariate statistics (Cox regression). Variables with *P*-values below 0.10 in the univariate analysis were further examined in the multivariate analysis. All statistical analyses were performed using EZR, which is a graphic user interface for R (The R Foundation for Statistical Computing, version 2.13.0).^11^*P-*values less than 0.05 were considered statistically significant.

## RESULTS

The baseline characteristics are shown in Table 1. Thiopurines were administered to 252 of 787 patients with UC (32.0%). The 161 patients included in the present study consisted of 139 patients (86.3%) who received AZA and 22 patients (13.7%) who received 6-MP. The initial doses were 0.84 ± 0.25 mg/kg/d (average, 46 ± 10 mg; range, 25–100 mg) and 0.52 ± 0.23 mg/kg/d (average, 29 ± 13 mg; range, 10–50 mg), respectively. The maintenance doses were 0.92 ± 0.31 mg/kg/d (average, 51 ± 15 mg; range, 25–100 mg) for AZA and 0.57 ± 0.23 mg/kg/d (average, 31 ± 13 mg; range, 10–70 mg) for 6-MP. Of the 161 patients, 17 (10.6%) were switched from tacrolimus to thiopurine therapy. Steroid therapy was administered to 153 patients (95.0%). Further, there were 122 steroid-dependent patients (75.8%) and 59 patients (36.6%) who had been treated with steroids at a dose of 40 mg/d or higher.

**TABLE 1.** Baseline Characteristics

	All (n = 161)
Male, number	92 (57.1%)
Age at onset, y	33 ± 16 (6–76)
Age at the start of thiopurines, y	39 ± 16 (15–80)
Extent at diagnosis	
Total colitis	118 (73.2%)
Left-sided colitis	42 (26.1%)
Proctitis	1 (0.6%)
Duration of UC, y	6.2 ± 7.1 (0.04–45.5)
Extraintestinal manifestations	15 (9.3%)
Current smoking	18 (11.2%)
Previous treatment	
Prior cytapheresis	43 (26.7%)
Prior corticosteroid	153 (95.0%)
History of treatment with a corticosteroid dosage ≥40 mg/d	59 (36.6%)
Induction treatment	
Concurrent use of mesalazine/salazosulfapyridine	158 (98.1%)
Corticosteroid	78 (48.4%)
Cytapheresis	16 (9.9%)
Tacrolimus	17 (10.6%)
Steroid dependence	122 (75.8%)
Steroid refractoriness	12 (7.5%)
Blood examination	
Leukocyte count (10^9^/L)	8.1 ± 3.0 (3.2–17.6)
Neutrophil count (10^9^/L)	5.6 ± 2.8 (1.8–15.5)
Hemoglobin (g/L)	12.1 ± 2.0 (7.6–16.4)
Mean corpuscular volume (fL)	87.3 ± 7.1 (67.0–106.5)
Platelet count (10^4^/L)	37.0 ± 13.4 (10.0–94.0)
C-reactive protein (mg/dL)	1.0 ± 1.9 (0.01–10.8)
Clinical outcomes	
CAI at the start of induction treatment	6.6 ± 3.2 (3–17)
CAI 1 y after induction treatment	3.5 ± 1.8 (2–10)
Exacerbation of UC	52 (32.3%)
Hospitalization for UC exacerbation	27 (16.8%)
CRC	4 (2.5%)
Colectomy	9 (5.6%)
Adverse events	10 (6.2%)
Concurrent disease	12 (7.5%)
Death	3 (1.9%)
Observation period, y	4.7 ± 4.3 (0.1–16.3)

**FIGURE 1.**
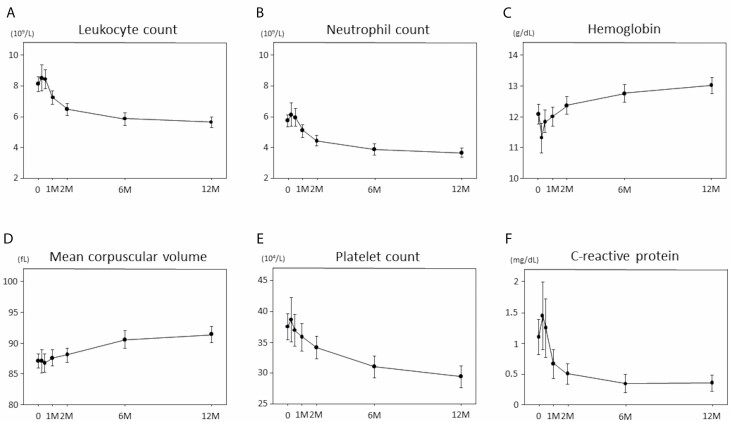
Transitional change of hematological examinations at weeks 0, 1, 2, 4, 8 and months 6, 12 after the start of induction therapy. A, Leukocyte; B, neutrophil; C, hemoglobin; D, mean corpuscular volume; E, platelet; F, C-reactive protein.

Based on the blood test data, the WBC and neutrophil counts started decreasing 2 weeks after the start of thiopurine therapy and had almost plateaued at 6 months. Hemoglobin levels reached the lowest level 1 week after the start of thiopurine therapy and slowly increased thereafter. The mean corpuscular volume started increasing 6 months after the start of thiopurine therapy. Lastly, the platelet counts started slowly decreasing 2 weeks after the start of thiopurine therapy (Fig. 1).

### Efficacy of Remission Maintenance Therapy With Thiopurines

**FIGURE 2.**
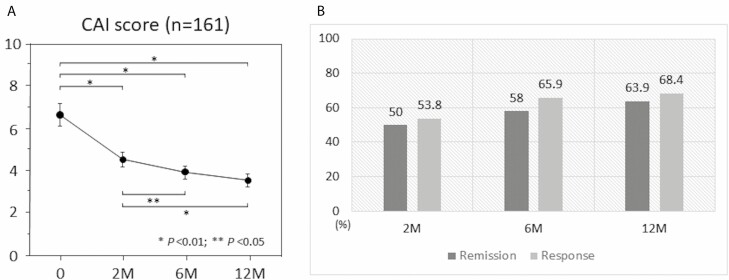
Clinical efficacy of thiopurine immunomodulators in total UC patients (n = 161). A, CAI of Lichtiger score at months 0, 2, 6, and 12 after the start of induction therapy. B, Clinical remission and response rate at months 2, 6, and 12 after the start of induction therapy.

**FIGURE 3.**
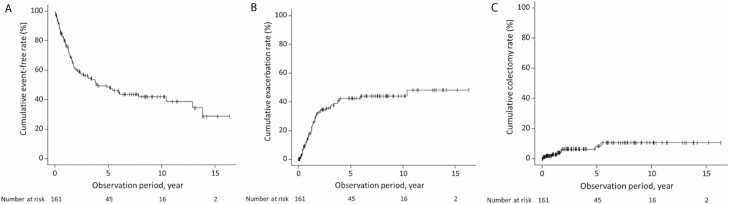
Kaplan–Meier analysis of total UC patients (n = 161). A, Cumulative event-free rate. B, Cumulative exacerbation rate. C, Cumulative colectomy rate.

The CAI was 6.6 ± 3.2 at baseline, 4.5 ± 2.1 at 2 months after the start of thiopurine therapy, 3.9 ± 1.8 at 6 months, and 3.5 ± 1.8 at 1 year, showing significant decreases from the baseline CAI (*P* < 0.001). At 2 months, 6 months, and 1 year after the start of thiopurine therapy, the clinical remission rates were 50.0%, 58.0%, and 63.9%, respectively, and the clinical response rates were 53.8%, 65.9%, and 68.4%, respectively (Fig. 2). At 1, 2, 5, and 10 years after the start of thiopurine therapy, the cumulative event-free rates were 77.6%, 60.8%, 48.5%, and 42.2%, respectively; the cumulative UC exacerbation rates were 17.0%, 32.5%, 42.2%, and 43.7%, respectively, and the cumulative colectomy rates were 0.6%, 1.3%, 8.5%, and 10.7%, respectively (Fig. 3).

### Investigation of UC Exacerbation During Remission Maintenance Therapy With Thiopurines

No significant differences were observed in sex, disease type, age at onset, or disease duration between the exacerbation and nonexacerbation groups. Among the 17 patients who were switched from tacrolimus to thiopurine therapy, there were more patients in the exacerbation group than in the nonexacerbation group (n = 9, 17.3% and n = 8, 7.3%, respectively). More patients were treated with tacrolimus in the exacerbation group (*P* = 0.09). Steroids had been administered at a dose of 40 mg/d or higher to 27 patients in the exacerbation group (51.9%) and 32 patients in the nonexacerbation group (29.4%). Significantly more patients had been treated with steroids at such a dose in the exacerbation group (*P* = 0.008) (Table 2). As for the risk for UC exacerbation during remission maintenance therapy with thiopurines, multivariate analysis identified the prior use of steroids at a dose of 40 mg/d or higher as a significant risk factor [hazard ratio, 2.26; 95% confidence interval (CI), 1.18–4.34; *P* = 0.014] (Table 3).

**TABLE 2.** Clinical Characteristics and Outcomes: Comparison Between the Exacerbation Group and the Nonexacerbation Group

	Exacerbation (n = 52)	Nonexacerbation (n = 109)	*P*
Male, number	29 (55.8%)	63 (57.8%)	0.87
Age at onset, y	31 ± 13 (6–76)	34 ± 17 (6–76)	0.27
Age at the start of thiopurines, y	37 ± 13 (15–67)	41 ± 18 (15–80)	0.16
Extent at diagnosis			0.07
Total colitis	33 (63.5%)	85 (78.0%)	
Left-sided colitis	18 (34.6%)	24 (22.0%)	
Proctitis	1 (1.9%)	0	
Duration of UC, y	5.8 ± 5.9 (0.1–24.4)	6.4 ± 7.6 (0.04–45.5)	0.65
Extraintestinal manifestations	4 (7.7%)	11 (10.1%)	0.78
Current smoking	6 (11.5%)	12 (11.0%)	1.00
Previous treatment			
Prior cytapheresis	19 (36.5%)	24 (22.0%)	0.05
Prior corticosteroid	49 (94.2%)	104 (95.4%)	0.71
History of treatment with a corticosteroid dosage ≥40 mg/d	27 (51.9%)	32 (29.4%)	0.008
Induction treatment			
Concurrent use of mesalazine/salazosulfapyridine	51 (98.1%)	107 (98.2%)	1.00
Corticosteroid	23 (44.2%)	55 (50.5%)	0.50
Cytapheresis	3 (5.8%)	13 (11.9%)	0.27
Tacrolimus	9 (17.3%)	8 (7.3%)	0.09
Steroid dependence	41 (78.8%)	81 (74.3%)	0.56
Steroid refractoriness	4 (7.7%)	8 (7.3%)	1.00
Blood examination			
Leukocyte count (10^9^/L)	8.2 ± 2.7 (4.1–17.6)	7.9 ± 3.0 (3.2–17.6)	0.71
Neutrophil (10^9^/L)	5.3 ± 2.2 (1.9–11.1)	5.7 ± 3.0 (1.8–15.5)	0.49
Hemoglobin (g/L)	11.8 ± 2.0 (7.6–15.2)	12.2 ± 2.0 (8.0–16.4)	0.25
Mean corpuscular volume (fL)	85.7 ± 6.6 (70.2–97.2)	88.0 ± 7.2 (67.0–106.5)	0.06
Platelet count (10^4^/L)	36.4 ± 10.6 (18.3–72.1)	37.3 ± 14.5 (10.0–94.0)	0.69
C-reactive protein (mg/dL)	0.9 ± 1.7 (0.01–9.0)	1.1 ± 2.1 (0.01–10.8)	0.62
Clinical outcomes			
CAI at the start of induction treatment	7.0 ± 3.4 (3–17)	6.5 ± 3.1 (3–16)	0.40
CAI 1 y after induction treatment	4.0 ± 2.2 (2–10)	3.3 ± 1.5 (2–10)	0.04
Hospitalization for UC exacerbation	24 (46.2%)	3 (2.8%)	<0.001
CRC	2 (3.8%)	2 (1.8%)	0.60
Colectomy	4 (7.7%)	5 (4.6%)	0.47
Adverse events	3 (5.8%)	7 (6.4%)	1.00
Concurrent disease	5 (9.6%)	7 (6.4%)	0.53
Observation period, y	4.9 ± 4.2 (0.2–14.9)	4.6 ± 4.4 (0.1–16.3)	0.70

**TABLE 3.** Predictive Factors of UC Exacerbation After Starting of Treatment With Thiopurines

	Hazard Ratio	95% CI	*P*
History of treatment with a corticosteroid dosage ≥40 mg/d*	2.26	1.18–4.34	0.014
Tacrolimus	1.44	0.57–3.63	0.44
Prior cytapheresis	1.34	0.68–2.65	0.40
Extent at diagnosis; proctitis^†^	6.40	0.77–53.4	0.09
Extent at diagnosis; total colitis^‡^	0.61	0.32–1.15	0.12
Mean corpuscular volume at the start of thiopurines	0.97	0.93–1.01	0.15

*Relative to the reference of history of no use of corticosteroids or corticosteroid dosage <40 mg/d.

^†^Relative to the reference of total colitis or left-sided colitis type.

^‡^Relative to the reference of left-sided colitis or proctitis type.

### Adverse Events Due to Thiopurines

**FIGURE 4.**
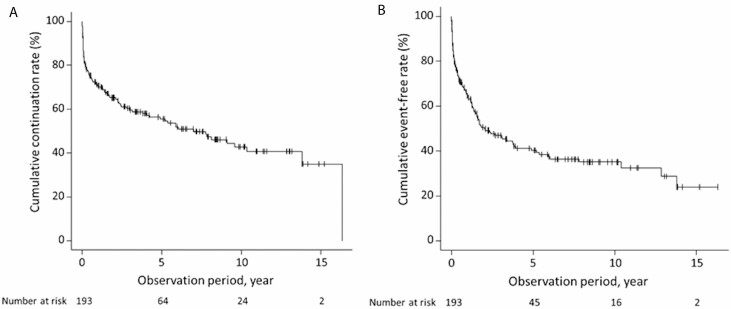
Kaplan–Meier analysis of UC patients including intolerance for thiopurine (n = 193). A, Cumulative continuation rate. B, Cumulative event-free rate.

Table 4 shows the adverse events observed in 193 patients, including 32 patients who were excluded from the main analyses because of the exclusion criterion of discontinuation of thiopurines due to intolerance within 3 months of thiopurine therapy. Adverse reactions occurred in 42 patients (21.8%), accounting for 46 events. In 32 patients developing adverse reactions within 3 months of thiopurine therapy, the main adverse reactions included liver dysfunction in 7 patients, nausea in 6 patients, fever in 4 patients, a WBC count of less than 3 × 10^9^/L in 3 patients, pancytopenia in 3 patients, and alopecia in 3 patients. Adverse reactions were observed in the remaining 10 patients beyond 3 months after the start of thiopurine therapy, including nausea in 3 patients, a WBC count of less than 3 × 10^9^/L in 3 patients, pancytopenia in 3 patients, alopecia in 1 patient, and drug induced eosinophilic pneumonia in 1 patient. The median time from treatment initiation to the onset of adverse events within 3 months and that beyond 3 months after the initiation of thiopurine therapy were 28 days (range, 7–91 days) and 1400 days (range, 148–3998 days), respectively. Concurrent diseases were observed in 18 patients (9.3%), including 10 patients with cancer [5 with colorectal cancer (CRC), 2 with ovarian cancer, 1 with basal cell carcinoma of the face, 1 with prostate cancer, and 1 with pancreatic cancer]. Of the 10 patients who developed cancer, the median age of cancer onset was 49 years, and 3 patients were 60 years or older, and no biologics were used during the observation period. Four patients died; the causes of death were UC exacerbation resulting in perforation and peritonitis (n = 1), pneumocystis pneumonia (n = 1), ovarian cancer (n = 1), and acute myocardial infarction (n = 1). In the 193 patients at 1, 2, 5, and 10 years after the start of thiopurine therapy, the cumulative continuation rates were 70.7%, 65.1%, 55.5%, and 41.9%, respectively, and the cumulative event-free rates were 64.5%, 50.6%, 40.3%, and 35.1%, respectively (Fig. 4).

**TABLE 4.** Adverse Events and Concurrent Disease

Adverse Events	(n = 46)	Concurrent Disease	(n = 18)
Myelotoxicity		Cancer	
Leucopenia	6 (3)	CRC	5
Neutropenia	1	Ovarian cancer	2
Pancytopenia	5 (2)	Pancreatic cancer	1
Gastroenterology and hepatology		Prostate cancer	1
Nausea	9 (3)	Facial basal cell carcinoma	1
Diarrhea	1	Infections	
Liver dysfunction	7	Pneumocystis pneumonia	1
Pancreatitis	1	Latent pulmonary tuberculosis	1
Hyperamylasemia	1	Perianal abscess	2
Allergic reactions		Purulent arthritis	1
Fever	4	Cellulitis	1
General malaise	2	Others	
Rash	3	Depression	1
Others		Myocardial infarction	1
Alopecia	4 (1)		
Drug induced eosinophilic pneumonia	2 (1)		

The numbers in parentheses are the number of patients developing adverse reactions beyond 3 months after the start of thiopurines.

### Withdrawal From Thiopurines

For causes other than UC exacerbation and adverse events, thiopurines were discontinued in 18 patients (9.3%) during the follow-up period. Fifteen patients were withdrawn from the drugs because they requested it. During the follow-up period, 5 of them started biologic therapy and subsequently withdrew from thiopurines. In 3 patients, treatment was interrupted for several months because of pregnancy and delivery and resumed afterward. Regarding the clinical courses after the withdrawal from thiopurines in the 15 patients, 9 patients maintained clinical remission with mesalazine alone; 1 patient started biologic therapy because of UC exacerbation; and 5 patients maintained remission with biologics. In the 9 patients who maintained clinical remission with only mesalazine after withdrawing from thiopurines, the mean duration of thiopurine therapy was 4.1 ± 5.3 years (range, 0.4–16.3 years; median, 0.9 years).

## DISCUSSION

### Long-Term Efficacy of Thiopurines

Thiopurines have been reported to have markedly beneficial effects on the course of UC and to reduce the risks of colectomy, hospitalization, UC exacerbation, and anti-TNF therapy initiation over 10 years after the start of thiopurine therapy.^12^ In our study, 77% of the patients had steroid-dependent UC, for which thiopurines were found to be effective for a long period of time. After approximately 4 years of thiopurine therapy, fewer patients experienced UC exacerbation, and thiopurines did not lose their efficacy, unlike biologics. Similar results were obtained for colectomy. The number of patients who underwent colectomy plateaued after approximately 5 years of thiopurine therapy. In a comparative study on remission induction with AZA and mesalazine in steroid-dependent UC, AZA was more effective in achieving clinical and endoscopic remission and for reducing steroid doses than mesalazine.^13^ Immunomodulators are effective for maintaining remission in UC^14,15^; in particular, AZA is effective for maintaining remission for a long period of time.^16^ In 71% of patients who were continuously treated with AZA for 6 months or more, AZA was continuously administered for 3 additional years.^17^ In a study on long-term AZA monotherapy for UC with a mean treatment duration of 5 years, a comparison between patients with and without mucosal healing showed that the duration of AZA monotherapy was approximately 2 times longer in the former than in the latter.^18^ Although AZA is effective for maintaining remission for a long period of time, the present study identified prior treatment with steroids at a dose of 40 mg/d or higher as a significant predictor of exacerbation during remission maintenance therapy with thiopurines. Thus, in such cases, therapeutic strategies should be considered in anticipation of introducing or switching to biologic therapy.

### Effect of Thiopurines on Preventing Colectomy

It is reported that thiopurines were a significant factor for the prevention of colectomy.^19^ In patients who did not switch to AZA after remission induction with cyclosporine or infliximab, the risk for colectomy was 8 times higher.^20^ Switching to AZA after remission induction with cyclosporine is effective for avoiding colectomy.^21^ The risk for colectomy was 71% lower in patients treated with thiopurines for at least 1 year than in those treated for 3 months or less.^22^ Also, in the present study, thiopurines prevented colectomy over a long period of time, and only a few patients who were continuously treated with thiopurines for 5 years or longer required colectomy.

### Adverse Events Due to Thiopurines

In our study, the incidence of adverse reactions to thiopurines was 22%, which was comparable with the rate that Yamada et al reported (28.8%).^23^ Of 46 adverse reactions, myelotoxicity accounted for 26%, intolerance accounted for 22%, and hepatotoxicity accounted for 15%. According to the breakdown of adverse reactions reported by Eriksson et al, hepatic dysfunction accounted for 23%, nausea for 23%, malaise for 27%, arthralgia for 10%, headache for 12%, and hypersensitivity for 4%.^24^ Yamada et al reported that myelotoxicity accounted for 29%, hepatic dysfunction for 24%, and nausea for 24%.^23^ The proportions of adverse reactions in our study were comparable with those presented in both reports. In a study which enrolled 396 patients with IBD, the observed toxicities of thiopurines included pancreatitis (3.3%), myelosuppression (2%), allergic reaction (2%), hepatic dysfunction (0.3%), infectious complication (7.4%), and malignant lymphoma (3%).^25^ While 2–3 months are generally required for thiopurines to exert their effects, adverse reactions to thiopurines occur shortly after treatment initiation or within the first 3 months of treatment. Adverse reactions rarely occur after more than 12 months.^12^ Because many serious adverse reactions occur at the early stage of treatment, frequent examinations and blood tests are necessary at the start of thiopurine therapy.

### Thiopurines and Carcinogenesis

During the study period in the present study, 5 patients developed CRC, and 5 patients developed cancer in other organs. Theoretically, immunomodulators may increase the risk of CRC via immunosuppression or prevent CRC by ameliorating chronic mucosal inflammation. Although a meta-analysis failed to find any significant chemopreventive effect of thiopurines (odds ratio: 0.87; 95% CI: 0.71–1.06),^26^ an observational cohort study indicated that patients with UC treated with long-term thiopurine therapy might have a lower risk of CRC than patients who are not treated with thiopurine therapy (hazard ratio: 0.28; 95% CI: 0.1–0.9).^27^ In Japan, approximately 10% of newly registered patients with UC are 65 years of age or older,^28^ and the number of elderly patients with UC is increasing. Aging of patients with UC is expected to continue.^29,30^ Elderly patients with IBD are at a high risk for malignancies unrelated to IBD.^31^ In addition, thiopurines may increase the risks of urinary tract cancer,^32^ acute myeloid leukemia, myelodysplastic syndrome,^33^ lymphoproliferative disease,^34^ and nonmalignant melanoma skin cancer.^35^ Thus, the chance of encountering cancer patients with IBD may increase. The European Crohn’s and Colitis Organization guidelines state that, when cancer develops in patients taking thiopurines, discontinuation of thiopurines is necessary—at least until the completion of cancer treatment—and that, given the risk for cancer recurrence, it should be considered to delay the resumption of immunosuppressive therapy for 2 years after the completion of cancer treatment.^36^

### Measures Against Adverse Events

Recently, thiopurines have been used in combination with allopurinol to reduce doses of thiopurines. In the present study, allopurinol was concomitantly used in 7 patients (3.6%) during the dose escalation of thiopurines. A combination of xanthine oxidase inhibitors and allopurinol is known to inhibit the metabolism of AZA, which results in extremely elevated blood levels of AZA. In patients intolerant to thiopurines, the concomitant use of allopurinol allowed dose reduction of thiopurines to 25% of the original dose. The response rates for adverse reactions observed before the use of allopurinol were 94% for hepatic dysfunction, 84% for nausea, 62% for malaise, 100% for arthralgia, 88% for headache, and 100% for hypersensitivity. All adverse reactions were markedly relieved.^24^ In a prospective study comparing thiopurines alone and in combination with allopurinol, the clinical remission rate at 24 weeks was significantly higher for the combination of thiopurines and allopurinol.^37^ The combination of thiopurines and allopurinol can reduce the hepatotoxicity of thiopurines,^38^ and the influenza-like symptoms hypothesized to be associated with excess levels of 6-thioinosine-5-triphosphate may be relieved by reducing doses of thiopurines through the concomitant use of allopurinol.^39^

### Withdrawal From Thiopurines

As for thiopurines, the appropriate duration of administration and timing of withdrawal are controversial issues. In the present study, 15 patients (7.8%) withdrew from thiopurines, and 9 of them remained in clinical remission with only mesalazine. The remission maintenance rate in IBD after the discontinuation of AZA was 63% at 1 year, 34% at 3 years, and 25% at 5 years.^40^ Factors affecting relapse after discontinuation of AZA were a lack of sustained remission during AZA therapy, total colitis type, and short duration of AZA therapy.^41^ Long-term administration of thiopurines is recommended.^42^ Hawthorne et al consider it preferable to continue AZA therapy for at least 2 years after achieving remission.^43^ However, treatment with potent immunomodulators is likely difficult to continue for a long period of time. Thus, discontinuation of treatment with immunomodulators in patients with IBD should be individually determined in consideration of the patient’s medical history and disease status, among other factors.^44^

The study limitations include the single-center retrospective cohort study design and the small sample size. Although thiopurine doses were adjusted to obtain a WBC count of 3–5 × 10^9^/L, dose adjustment was left to the discretion of the attending physicians.

## CONCLUSIONS

This study confirmed that thiopurines were effective for maintaining remission over a long period in patients with steroid-dependent/refractory UC. However, patients taking thiopurines over a long period of time should be closely monitored for risks of infection and carcinogenesis. Thus, the future issues appear to be the timing of withdrawal from thiopurines and profiling of patients who can withdraw from thiopurines.

## Data Availability

Data not publicly available.

## Notes

Funding: All authors have no support or funding to report.
